# Complex Hospital-Based Electronic Prescribing–Based Intervention to Support Antimicrobial Stewardship: Qualitative Study

**DOI:** 10.2196/54458

**Published:** 2024-07-26

**Authors:** Kathrin Cresswell, Susan Hinder, Aziz Sheikh, Neil Watson, David Price, Andrew Heed, Sarah Katie Pontefract, Jamie Coleman, Jillian Beggs, Antony Chuter, Ann Slee, Robin Williams

**Affiliations:** 1 Usher Institute University of Edinburgh Edinburgh United Kingdom; 2 Newcastle upon Tyne Hospitals National Health Service Foundation Trust Newcastle United Kingdom; 3 Institute of Clinical Sciences University of Birmingham Birmingham United Kingdom; 4 Institute for the Study of Science, Technology and Innovation The University of Edinburgh Edinburgh United Kingdom

**Keywords:** antimicrobial stewardship, electronic prescribing, evaluation, healthcare, qualitative study, hospital-based, electronic prescribing, e-prescribing, prescribing, prescription, ePAMS+, antimicrobial resistance, AMR, complex intervention, complex interventions, educational, behavioral, technological, public health, implementation, AMS, hospital, hospitals, development, in-depth, interview, interviews, observation, observations, prescriber, prescribers, nurse, nurses, pharmacist, pharmacists, microbiologist, microbiologists, thematic analysis, antimicrobial, antimicrobials

## Abstract

**Background:**

Antimicrobial resistance (AMR) represents a growing concern for public health.

**Objective:**

We sought to explore the challenges associated with development and implementation of a complex intervention designed to improve AMS in hospitals.

**Methods:**

We conducted a qualitative evaluation of a complex AMS intervention with educational, behavioral, and technological components in 5 wards of an English hospital. At 2 weeks and 7 weeks after initiating the intervention, we interviewed 25 users of the intervention, including senior and junior prescribers, a senior nurse, a pharmacist, and a microbiologist. Topics discussed included perceived impacts of different elements of the intervention and facilitators and barriers to effective use. Interviews were supplemented by 2 observations of ward rounds to gain insights into AMS practices. Data were audio-recorded, transcribed, and inductively and deductively analyzed thematically using NVivo12.

**Results:**

Tracing the adoption and impact of the various components of the intervention was difficult, as it had been introduced into a setting with competing pressures. These particularly affected behavioral and educational components (eg, training, awareness-building activities), which were often delivered ad hoc. We found that the participatory intervention design had addressed typical use cases but had not catered for edge cases that only became visible when the intervention was delivered in real-world settings (eg, variations in prescribing workflows across different specialties and conditions).

**Conclusions:**

Effective user-focused design of complex interventions to promote AMS can support acceptance and use. However, not all requirements and potential barriers to use can be fully anticipated or tested in advance of full implementation in real-world settings.

## Introduction

Antimicrobial resistance (AMR) is a serious public health threat [1]. It involves bacteria becoming resistant to antibiotics, potentially leading to infectious diseases no longer being treatable with antimicrobial agents and is driven by antibiotic use [2,3]. The patient, health care, and economic costs of AMR are significant. Some estimates indicate that over 10 million deaths worldwide will be due to AMR by 2050, with significantly higher morbidity in resistant bacteria than nonresistant bacteria, costing US $1 trillion annually [4].

AMR is exacerbated by suboptimal prescribing practices, including, for example, over-prescribing of antibiotics, incomplete treatment courses, incorrect dosages or durations, and inadequate diagnosis and testing [5]. Antimicrobial stewardship (AMS) is an approach that attempts to refine and reduce inappropriate antibiotic use by improving prescribing and review practices [6]. Many AMS programs have targeted hospitals, as these are known to have high rates of inappropriate use of antibiotics [7]. Complex interventions with multifaceted, interconnected educational, behavioral, and technological components have the potential to enhance AMS [8]. Here, evidence suggests that behavioral change AMS interventions may encourage good AMS practice and adherence to guidelines by health professionals. However, there is a lack of good-quality studies, especially as behavioral components and contexts vary [9,10]. There is some evidence that adherence to guidelines in AMS interventions can result in reductions in mortality [7]. The cost-effectiveness of AMS programs is also still somewhat uncertain [11]. Clinical decision support and computerized provider order entry systems are associated with appropriate antimicrobial therapy, but impact on mortality is mixed [12,13]. This may be due to systems not integrating effectively with organizational environments, contexts of use, and existing technological systems [14].

However, the mechanisms of complex interventions are often hard to trace and attribute in quantitative studies, which is why qualitative work can be particularly helpful in exploring the processes of complex interventions consisting of multiple components [15]. Qualitative work can also explore unanticipated consequences and reasons for why anticipated outcomes may not materialize [16,17]. These issues can be exacerbated in digitalization initiatives, where technological components of interventions need to be woven into an interrelated and rapidly evolving technical infrastructure and workflows, resulting in distributed effects that are often part of wider organizational transformations [18].

In attempts to address this issue, qualitative studies accompanying quantitative assessments have been promoted as a way to help assess contextual variations, processes, and mechanisms of action [19]. In addition, there have been increasing efforts to assess whether the intervention was delivered as intended through quantitative means (eg, by developing fidelity indices) [20,21]. However, although the importance of context is increasingly understood in complex AMS interventions [22-24], there is still a lack of work exploring organizational environments, existing technologies, and contexts surrounding the promotion of AMS practices. For example, a recent study comparing a complex AMS intervention in 2 hospitals with the same digital system found that factors including high-level support, resources, and interdepartmental relationships were crucial factors in determining use of the intervention [22].

What is needed to progress are detailed insights into the challenges associated with the development, evaluation, and implementation of complex interventions. These are, at present, notably absent from the empirical literature [25], which emphasizes carefully curated accounts of successful measurements.

Here, we sought to address this gap by exploring how an e-prescribing–based complex AMS intervention was developed and implemented to extract lessons for the implementation and evaluation of complex digitalization initiatives in health care settings.

## Methods

### Intervention

We conducted a qualitative study of the early use of an e-prescribing–based complex AMS intervention that was integrated within a commercial e-prescribing system and deployed in an acute hospital in England. The hospital served a patient population of over 3 million and had achieved Healthcare Information and Management Systems Society (HIMSS) Level 6 at the time of data collection in 2022.

**Figure 1:**
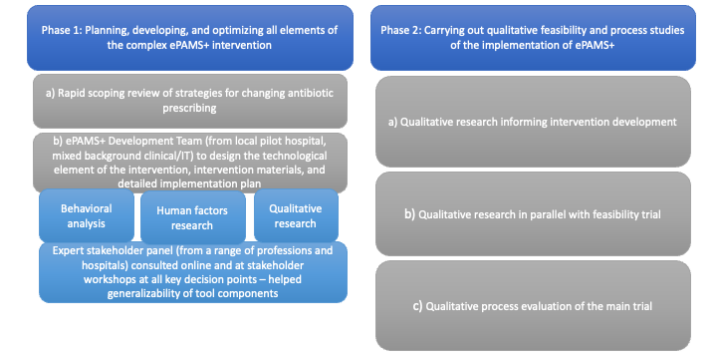
Overview of the qualitative work. ePAMS+: electronic Prescribing-Based Antimicrobial Stewardship Plus.

This study was part of a larger qualitative process evaluation of the development, feasibility trial, and main full-scale trial of the intervention. This paper is mostly reporting on the implementation aspect, although indirectly affecting the (future) development of electronic Prescribing–Based Antimicrobial Stewardship Plus (ePAMS+) through the feasibility trial findings. Figure 1 illustrates the qualitative work. The intervention was developed in Phase 1, and here we report on Phase 2b, a qualitative process evaluation of the feasibility trial [26]. The integrated mixed methods evaluation of that trial will be reported elsewhere.

**Figure 2:**
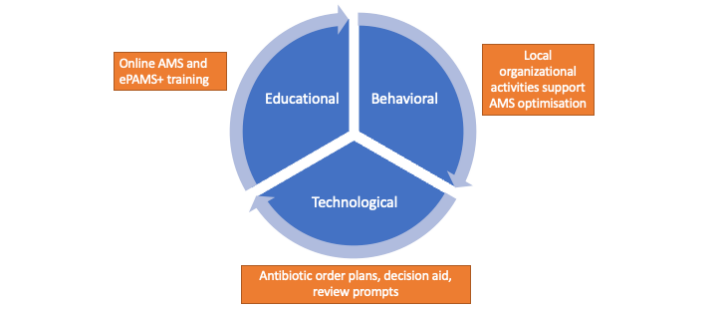
Diagram of the electronic Prescribing-Based Antimicrobial Stewardship Plus (ePAMS+) intervention. AMS: antimicrobial stewardship.

The developed intervention was called ePAMS+. Its technological and behavioral and educational components are summarized in Textbox 1 and Figure 2. The logic model summarizing the individual components is provided in a paper describing the intervention development process [27].

**Key elements of the electronic Prescribing–Based Antimicrobial Stewardship Plus (ePAMS+) intervention.**

**Technological components**

- Additional method of prescribing antibiotics (not compulsory) on the electronic prescribing system
- Antibiotic ordering plan
  Dose, route, frequency
  Duration
  Indication
  Body system
  Diagnostic confidence: possible risk, probably infection, prophylaxis
  Laboratory orders
- Single antibiotics (not protocols)
- Pharmacy antibiotic review
- Cultural and sensitivity test results
- Alert to undertake antibiotic review after 48 hours (passive)
  Not active evenings and weekends when on-call doctors are too busy
  Triggers when opening patient record if review overdue
  Can be overridden
- Antibiotic review documentation: cultures reviewed, senior clinician review, intravenous to oral switch, finalized prescription

**Educational and behavioral components**

- Online antimicrobial resistance (AMR) and ePAMS+ training (approximately 30 minutes of learning)
- Hosted online using a National Health Service (NHS)–approved URL [28]
- The online module covers the following learning outcomes:
  List the factors to consider when initiating antibiotics, switching the route of administration, and stopping treatment.
  Discuss the risks of staying on antibiotics for longer than is clinically needed.
  List the tools provided by the ePAMS+ intervention in this hospital.
  Explain how order plans work and how these have been set up to aid your decision-making.
  Explain the components of the Antibiotic Review Kit decision aid and how these can encourage good stewardship within ePAMS+.
- Pretest and posttest so learners can assess their baseline knowledge and knowledge acquisition
- Videos of the ePAMS+ tool to explain to learners how the tool is accessed and used in practice

In addition to the online training, it included ad hoc face-to-face training by study lead clinicians.

### Ethics Approval

Ethical approval for the qualitative research was obtained from the North of Scotland Research Ethics Service on February 6, 2020 (reference 19/NS/0174). Ethical approval for the feasibility trial and quantitative analysis was granted by the London South East Research Ethics Committee on April 25, 2022 (reference 22/LO/0204).

### Sampling and Recruitment

The senior e-prescribing pharmacist in the hospital developed the technical aspects of ePAMS+ in collaboration with the research team, drawing upon wider stakeholder engagement [27]. Their gatekeeper role helped us to establish contact with clinical leads and early users of the intervention. We selected 5 acute wards with high antibiotic use for the feasibility trial to be first adopters of ePAMS+. These included Infectious Diseases, Elderly Care, 2 Oncology wards, and Respiratory Diseases. We excluded wards that had high prophylactic use rates of antibiotics (eg, general surgical wards), as the indication for antimicrobial prescribing differed to that in medical wards.

We sampled individual study participants based on their use and knowledge of ePAMS+ (ie, those who had planned the intervention and implementation and those who were current or anticipated users). In recruitment, we worked closely with the clinical lead (an Infectious Disease consultant). We also interviewed the clinical lead and the e-prescribing pharmacist to gain insights into delivery processes. Sampling was opportunistic and ad hoc when the lead researcher (SH) visited each ward (intense work pressures for the hospital prevented planned interview schedules). We took every opportunity to speak to ePAMS+ users about their experiences and observe them using the intervention. We used purposive sampling to get a variety of viewpoints from a range of different professions of varying levels of seniority. We also used snowball sampling, asking participants if they could recommend further interviewees, looking for varied experiences (both positive and negative).

### Data Collection

We explored prescribers’ experiences using ePAMS+ 2 weeks and 7 weeks after “go live,” and SH collected qualitative data during 2 week-long field visits in October 2022 and December 2022.

Interviews explored existing AMS practices, perceived impacts of different elements of ePAMS+, facilitators and barriers to effective use, and suggested improvements (Textbox 2). Questions were tailored to emerging findings and the role of the interviewee, and we also fed emerging findings into concurrent data collection, which resulted in some modifications to the topic guide as the study progressed. They were digitally audio-recorded and professionally transcribed.

**Detailed topic guide interviews.**

- What is your role?
- Have you been involved in the development and delivery of the electronic Prescribing–Based Antimicrobial Stewardship Plus (ePAMS+) intervention?
- What is your understanding of the ePAMS+ intervention?
- How has training been delivered?
- Did you do the training module?
- Is there anything you would change in the training?
- In your opinion, what are the positive and negative aspects of ePAMS+?
- How well does ePAMS+ promote antimicrobial stewardship in your hospital?
- In your opinion, how does the intervention affect workflow, processes, and relationships of health care professionals?
- How might the design/usability/intelligibility of the ePAMS+ intervention be improved?
- Is there anything that you would have done differently in implementing ePAMS+?
- What guidelines are in place? Which guidelines do you use generally?
- What are the practices of antimicrobial stewardship in your ward/hospital?
- What barriers to antimicrobial stewardship do you perceive there to be in your ward/ hospital? In general?
- If you could, is there any aspect of the review process that you would want to change?
- If you were to describe an “ideal” intervention for antimicrobial stewardship that would combine the e-prescribing system and work practices, what would it look like?
- How has the intervention changed your antimicrobial prescribing, if at all?
- Is there anything you would like to change/add to the AMS intervention.
- Are there any other changes that would improve antimicrobial prescribing?

Where feasible, we also conducted nonparticipant observations of ePAMS+ use (Textbox 3). This involved the researcher (SH) observing ward rounds and clinical discussions before and after these (including handovers). The researcher took notes during these meetings, including describing the setup, issues discussed in relation to AMS, and emerging tensions. We did not record any patient-specific data. We stopped data collection when we reached thematic saturation, that is, when no new substantive themes were emerging from concurrent data collection in both interviews and observations [29].

**Observation recording sheet.**

- Place of observation
- Description of place
- Person observed
- Role
- Person asked if they have heard of, trained for, or used electronic Prescribing–Based Antimicrobial Stewardship Plus (ePAMS+)
- Person interviewed
- Role
- Activity observed
- Notes on non-audio-recorded comments to researcher
- Interaction with researcher
- Notes on observed conversations
- Reflections of researcher
- Further research questions

### Data Analysis

Interview transcripts and observation notes were uploaded to NVivo (v.12, QSR International). SH did the initial coding using the dimensions of the Technology, People, Organizational, and Macroenvironmental (TPOM) as the coding framework [30]. We also allowed for any additional themes arising and paid particular attention to emerging issues. We conceptualized the hospital as a case and explored emerging impacts, user experiences, and variations in contexts, paying specific attention to tensions and differences in perspectives. We fed these back to the ePAMS+ development team.

As the focus of our analysis was on assessing the intervention design and on exploring the delivery of the intervention, we extracted relevant themes in relation to planning, intervention content, delivery, implementation, and adoption. These were further refined in interactive discussion workshops with social scientists in the team (SH, KC, and RW). All researchers leading the data collection and analysis were social scientists by background and were also involved in developing the intervention.

## Results

### Participants

We interviewed 23 junior doctors, 2 consultants, 1 senior nurse, the e-prescribing pharmacist who developed the ePAMS+ intervention, and 1 microbiologist over 2 site visits (Table 1).

**Table 1:** Participant characteristics.

Participant number	Role	Gender	Age range (years)	Ward	Go live site visit 1	Length of interview 1 (minutes)	Go live site visit 2	Length of interview 2 (minutes)
1	Consultant	Male	40-50	Hematology/Oncology	November 1, 2022	27	N/A^a^	N/A
2	Registrar/ST3 Specialty trainee	Female	30-40	Infectious Diseases	October 27, 2022	26	N/A	N/A
3	Junior doctor, Foundation Year 2	Male	20-30	Infectious Diseases	October 27, 2022	29	November 28, 2022	15
4	Registrar	Female	30-40	Infectious Diseases	October 25, 2022	7	N/A	N/A
5	Senior nurse	Female	40-50	Infectious Diseases	October 25, 2022	24	N/A	N/A
6	Registrar/ST5 Specialty trainee	Male	30-40	Infectious Diseases	October 27, 2022	12	December 2, 2022	8
7	Pharmacist (ePAMS+^b^ developer)	Male	50-60	Pharmacy	November 2, 2022	60	N/A	N/A
8	Junior doctor, Foundation Year 2	Female	20-30	Hematology/Oncology	November 2, 2022	5	December 1, 2022	3
9	Registrar	Female	30-40	Hematology/Oncology	November 2, 2022	5	N/A	N/A
10	Junior doctor, Foundation Year 2	Female	20-30	Hematology/Oncology	November 2, 2022	9	N/A	N/A
11	Consultant	Male	40-50	Microbiology	November 3, 2022	40	N/A	N/A
12	Registrar/ST5 Specialty trainee	Male	40-50	Infectious Diseases	October 27, 2022	12	N/A	N/A
13	Registrar/ST 5 Specialty trainee	Male	40-50	Emergency Department Assessment Suite	November 9, 2022	17	N/A	N/A
14	Internal Medicine Trainee Year 1	Female	40-50	Elderly Care	November 9, 2022	3	N/A	N/A
15	Internal Medicine Year 2	Male	30-40	Respiratory	November 9, 2022	21	N/A	N/A
16	Junior doctor, Foundation Year 1	Male	20-30	Respiratory	November 9, 2022	21	N/A	N/A
17	Consultant	Male	40-50	Infectious Diseases	November 2, 2022	27	N/A	N/A
18	Registrar Specialty Trainee A&E^c^	Female	40-50	Emergency Department Assessment Suite	N/A	N/A	November 28, 2022	7
19	Internal Medicine Trainee Year 3	Female	30-40	Emergency Department Assessment Suite	N/A	N/A	November 28, 2022	5
20	Core Trainee Year 1	Male	30-40	Emergency Department Assessment Suite	November 9, 2022	24	N/A	N/A
21	Internal Medicine Trainee 1 (locum)	Male	30-40	Haematology/Oncology	N/A	N/A	November 30, 2022	21
22	Junior doctor, Foundation Year 2	Female	20-30	Emergency Department Assessment Suite	N/A	N/A	December 1, 2022	17
23	Registrar/Speciality Trainee	Male	30-40	Emergency Department Assessment Suite	N/A	N/A	December 1, 2022	7
24	Internal Medicine Year 2	Male	30-40	Emergency Department Assessment Suite	N/A	N/A	December 2, 2022	8

^a^N/A: not applicable.

^b^ePAMS+: electronic Prescribing–Based Antimicrobial Stewardship Plus.

^c^A&E: Accident and Emergency.

### Intervention

The implemented ePAMS+ intervention consisted of the following elements (Textbox 2): (1) a technological component, (2) development of an online educational training tool, (3) behavioral change components.

Overall, AMS was viewed as important with potential for improvement for prescribers:

It definitely makes you think about what you’re prescribing and why a little bit more because there are a few more steps involved in it...It’s good to see all the antibiotics in one place, rather than having to type in different names, so just typing in ePAMS, you can see all the different antibiotics. It’s obviously got prompts for using the [hospital] guidelines and things like that.

Participant 19, Female, Registrar

Use of ePAMS+ was voluntary during the feasibility trial but was planned to be made compulsory in the future. It was designed to be easy to use, bringing up a list of commonly prescribed antibiotics and triggering an antibiotic review pop-up 48 hours after prescribing.

**Figure 3:**
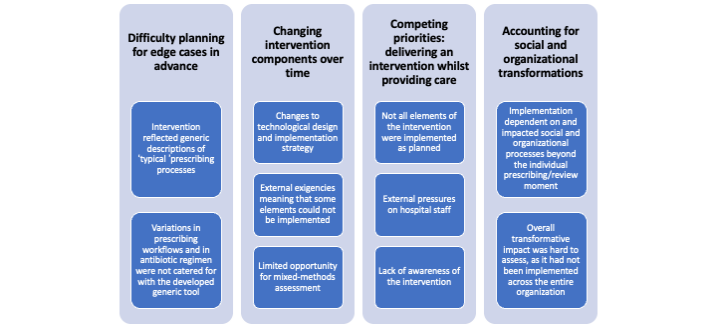
Overview of identified themes and subthemes.

Overall, we found several factors affecting the delivery of the intervention, including evolving technological properties, social and organizational transformations that could not be planned, and external pressures on hospital operations (Figure 3, Multimedia Appendix 1). These will be explained in more detail in the following paragraphs.

### Difficulty Planning for Edge Cases That Did Not Fit Within “Typical” Prescribing Processes in Advance

ePAMS+ was designed based on extended requirements elicitation with experienced health care professionals. This had generated descriptions of “typical” prescribing processes from the perspective of some specific individuals and contexts. The feasibility trial of the intervention in real-world settings identified a wider range of use processes and contexts than had been identified by stakeholder engagement [27]. Variations in prescribing workflows and in antibiotic regimen were not catered for with the developed generic intervention. This highlights the difficulties for stakeholders in anticipating the significant range of use cases, contingencies, and needs in requirement elicitation exercises, which consequently tend to focus on generic rather than “edge” cases.

What a focus group tells you and what a senior clinician tells you, and what a junior doctor wants is sometimes quite different, so we originally designed it with a link to the guidelines in but the senior clinician said there’s no point in having that because at that point you already know what antibiotic you want to prescribe so why would you need to go into the guideline but then...I think what senior clinicians were thinking about that’s the initial decision...but I think what they weren’t thinking about the purpose of the plan is to also allow review.

Participant 7, Male, Clinical Informatics Pharmacist

For example, ePAMS+ listed only commonly prescribed antibiotics and followed a generic process for administration and review. However, some antibiotics (eg, gentamicin) only had single doses, so the 48-hour prompt for review was not relevant.

The whole idea of a forty-eight-hour review is completely meaningless because it only ever is a one-off dose.

Participant 1, Male, Consultant

Similarly, for some antibiotics the first dose was different from the following doses, which ePAMS+ had difficulty accounting for.

[...] Doxycycline, you often will give like loading dose, and then give a lower dose the next few days, but ePAMS will only prescribe the loading dose.

Participant 15, Male, Specialty Trainee Year 2

ePAMS+ also did not list all the antibiotics used in the hospital. This made it difficult to prescribe combinations of antibiotics, as ePAMS+ was not set up to support complex antibiotics plans with combinations of antibiotics.

So it's not got every antibiotic on. I was trying to use it on assessments for a couple of more challenging patients with complex antibiotic plans. And it's a bit...what I did find a little bit challenging is sometimes they were going for a combination of antibiotics. One antibiotic is on ePAMS and the other one isn't. And I was less sure what to do in that situation. I just used it for one of them and not the other.

Participant 12, Male, Registrar

### Changing Intervention Components Over Time

The evolving properties of the trial intervention were partly due to our formative evaluation strategy, which included feedback to ePAMS+ developers and implementers (managers responsible for implementation) after data collection. These resulted in changes to technological design and implementation strategy. For example, during our field work, it was decided by the study lead consultant to include the Assessment Unit (a ward holding patients after Accident and Emergency, where decisions are made whether to admit to the hospital or discharge them home) due to the large volume of antibiotics prescribed there.

The intervention had been developed in close collaboration with key stakeholder groups [27]. However, some elements of the feasibility trial could not be implemented because of external exigencies, particularly as the hospital struggled to cope with significant workloads and pressures associated with the COVID-19 pandemic. This also meant that behavioral and educational components (including the educational component and implementation plans) were difficult for us to assess in a meaningful way, as we were only able to gain secure access to stakeholders opportunistically, at times when they were available.

In addition, not all the requirements identified in intervention development meetings were able to be implemented in the initial adoption for the feasibility trial, with some being held back for later adoption. The stakeholder groups described that initial prescribing of antibiotics was rarely done using indication-based order sets and instead used empiric single or multi-agent prescriptions. They deemed that order set–based ePAMS+ was not needed for initial use but could be developed for selected areas. Clinicians could still use the usual “Medications” screen to prescribe antibiotics—and many continued to do this, overlooking the ePAMS+ pathway. It was not possible to make the use of the ePAMS+ pathway mandatory in trial wards without compromising inpatient antibiotic prescribing in the wider organization and in outpatient settings; therefore, the option to use the more familiar pathways remained available to users.

[...] we’ll get to a point where [name] will say, we’ve gone round all of the wards, they’re using the ePAMS plan, now just switch it on for everywhere, if that makes sense, once people have got a bit more familiar with it, then those fields will just become mandatory.

Participant 7, Male, Clinical Informatics Pharmacist

### Competing Priorities: Delivering an Intervention While Providing Care

We found that not all elements of the intervention were implemented as planned. ePAMS+ was launched within an environment with multiple exigencies and competing pressures impacting the implementation uptake. The hospital had been working on an Operational Pressures Escalation (Opel) Level 4 (just after the launch of the intervention), driven by high levels of bed occupancy and increasing patient numbers attending Accident and Emergency. Opel levels assess the strain, demand, and burden experienced by hospitals. Level 4 denotes that the hospital faces significant challenges that impact patient safety [31]. The impending winter further exacerbated the pressure on staff. This contributed to ePAMS+ leads and adopters being extremely busy.

So I wouldn’t use it for that, especially if, you know, I’m stressed, I’m tired, if it’s out of hours, if someone’s really, really sick, I’m just going to do one, what my habit is, which is to go into medication list and two, just the safe option is to look for that order set so I know that I’m not going to miss anything. It’s going to prompt me and ePAMS doesn’t give me that.

Participant 8, Female, Junior Doctor Foundation Year 1

As a result, AMS in general and the ePAMS+ intervention specifically could not always be prioritized by implementers (ie, those managing the introduction of the intervention) and adopters (ie, end users of the intervention), and the lack of mandatory use meant that it could be ignored by potential adopters. This was particularly true for the educational and behavioral aspects of the intervention, which were often delivered opportunistically. ePAMS+ was implemented through a strategy of incremental adoption, allowing adopters to learn about ePAMS+ in an ad hoc manner and allowing optional use, rather than imposing compulsory use with “hard stops.” For example, ePAMS+ training was often delivered through peers or clinical champions, rather than being taken up through the online training platform developed as part of the intervention. Training videos were viewed as unlikely to be accessed by doctors working in the current busy working environment of the National Health Service (NHS).

So, somebody showed me how to prescribe on ePAMS, on the ward, like for five minutes and we just clicked the boxes and he showed me how to do it. But because no-one showed me how to do it, when I get the prompt [for review]...I’m quite busy, so pressing buttons and trying to figure out how to do it, isn’t at the top of my priority list.

Participant 10, Female, Junior Doctor Foundation Year 2

We also observed a general lack of awareness of ePAMS+ as a designated intervention to improve AMS among staff, which may have reflected the competing pressures for the attention of staff.

I’m aware of it. I believe it’s a tool to sort of try and promote antimicrobial stewardship, but I’ve not had any real sort of direct training or knowledge of it.

Participant 6, Male, Specialty Trainee Year 5

### Accounting for Social and Organizational Transformations

We further observed that the feasibility trial of ePAMS+ was dependent on and impacted social and organizational processes beyond the individual prescribing/review moment. Anticipated processes surrounding the use of ePAMS+ differed from emergent actions of users when the intervention was implemented in real-world settings. Here, we observed that the intervention changed the workflows of many different professions and across many different areas. The overall transformative impact was hard to assess, as it had not been implemented across the entire organization. For example, many ePAMS+ users moved around the organization, spanning settings where ePAMS+ was available and settings where it was not yet implemented. This was particularly visible in the Assessment Unit. If a doctor in the Assessment Unit had not used ePAMS+ to initiate a prescription, then it was not appropriate for the doctors on the ward the patient was being transferred to start an ePAMS+ care plan.

Because the problem is if you don’t do that, the patient goes from here, the Assessment Unit, to Ward 52, and they haven’t had it done under ePAMS, that means Ward 52 have to re-prescribe under ePAMS, and by then it might be 24 to 48 hours into admission, so the antibiotic doesn’t get reviewed until three to five days instead of two to four days, or roughly, you know, two to three days, so that’s a bit of a problem.

Participant 1, Male, Consultant

Efforts to incorporate understanding of social and organizational processes in designing and implementing the ePAMS+ intervention were well-received by users. For example, it was appreciated that the antibiotic review alerts were not issued at nights nor weekends when junior doctors were very busy covering multiple wards and often would not know the patient. An antibiotic review at those times would create a lot of work for a junior doctor when a more senior doctor was not available to provide guidance.

It prompts the doctor during working hours, when it’s not the weekend, to review the antibiotics, that's a huge advantage...because there aren't as many doctors around, and therefore, we wouldn't want, for instance, someone who's not familiar with a patient to have to make that decision. Also, it's a bit unfair too...because that will create a lot of work, increase the workload for a junior doctor. Because it'll probably be the [junior doctors] covering multiple wards to have to review ten, 20 antibiotics, and making a decision around antibiotics shouldn't really fall to the most junior person on the team.

Participant 13, Male, Specialty Trainee Year 5

## Discussion

### Summary of Findings

We undertook a qualitative evaluation accompanying a feasibility trial and used the results to trace how a complex e-prescribing–based intervention was delivered in a hospital setting. We found that the delivery of the intervention components was difficult to track, as implementers had to adjust implementation strategies to suit emerging local needs and priorities. This meant that behavioral and educational components were often delivered inconsistently across settings and in ways other than intended by the research team. We further observed that the intervention did not sufficiently account for variations in workflows, which only emerged when the intervention was implemented and tested in real-world settings.

### Strengths and Limitations

We produced an empirical account of how a complex e-prescribing–based intervention consisting of educational, behavioral, and technological components was delivered and received in a hospital setting. Voluntary adoption in 5 wards was intended to assess the feasibility of implementation and allow for learning to enhance both ePAMS+ design and implementation strategy. A hospital-wide implementation is planned to follow the feasibility work, and this will include hospital-wide mandated use of the intervention. It is likely to reveal additional complexities that a feasibility trial could not explore, particularly insofar as alternative prescribing pathways to ePAMS+ will no longer be available. A qualitative process evaluation of a potential main trial is planned.

However, due to the pandemic and other health service pressures, the feasibility work was undertaken at a later stage of intervention development than originally intended. In line with the person-based approach [32], this work would ideally have been undertaken before the feasibility trial so that emerging problems could have been detected and ironed out at an earlier stage. In addition, due to intense competing pressures in the hospital, we did not have access to observations of direct ePAMS+ use, although we did have the opportunity to get insights into AMS practices during observations of ward rounds. In our data collection, we did not manage to speak to any user who had completed the entire review process using the ePAMS+ intervention. This was in part due to the limited scale (5 wards) and period for which the intervention had been live at the time of data collection. This will be redressed in the planned hospital-wide roll-out of the intervention when we hope also to be able to observe how the intervention becomes embedded in routine practice. The full implementation is also likely to include a refined intervention based on these experiences. Nevertheless, our key contribution is a unique insight into how an intervention is developed and implemented in practice.

### Integration of Findings With the Existing Literature

This work supports the limited existing literature surrounding the processes and contexts in the delivery of multifaceted interventions seeking to promote AMS [18,22-24]. In doing so, we have illustrated that not only organizational factors are crucial in driving the implementation and adoption of AMS interventions but also practices vary among organizational units that in turn influence the adoption of the intervention in an organizational system. We have also shown how external pressures can impact implementation and adoption.

Our findings raise issues around measuring impacts of interventions and the ability to detect these. Interventions are delivered not in a static environment but rather under severe competing external pressures that may have a significant impact on adoption and on measurable outcomes. Our qualitative evaluation accompanying a feasibility trial highlighted these, although quantitative approaches to evaluation often neglect these dimensions, assuming that interventions are implemented in static environments according to predetermined plans [33]. Although there is now an increasing focus on mixed methods work exploring both intervention processes and outcomes [19], honest accounts of challenges encountered during intervention delivery are rare. Most existing work focuses on exploring barriers and facilitators to “success” from managerial perspectives [34], as opposed to surfacing tensions and trade-offs that must be made when designing and delivering complex multifaceted interventions in health care delivery environments. Rolling out an intervention in complex settings with local competing priorities surrounding care delivery may mean that it is not implemented as envisaged. Although competing priorities have been widely documented in the literature [25], it is unclear precisely how they impact delivery. We have added to the debate, discussing how behavioral and educational components (including training) may be delivered flexibly.

We have also illustrated how intervention design differs from its real-life application. We cannot assume that developed intervention logic models are faithfully translated into practice, as they are unlikely to consider the variety of use cases in actual practice. This has implications for traditional approaches to documenting interventions [35]. It also has implications for technological development of applications that are used by a variety of stakeholder groups. If a technology is tailored to particular use cases, this may impair its usability and use across a wider range of workflows and specialist users [36].

Our study has further shown how complex interventions are not delivered in a vacuum and that intervention planning needs to consider the socio-organizational contexts in which systems are deployed [37]. Although these are difficult to anticipate in advance, they have significant impacts on patterns of use, particularly in interventions with technological components that span professional boundaries [38].

### Implications for Policy and Practice

We give an overview of lessons learned from this work that can be applied to future implementation efforts in Textbox 4.

**Lessons learned from this work.**

- Exercise care in choice of when and where to introduce complex interventions (eg, avoid periods with severe internal [eg, winter] or internal [eg, during electronic health record (EHR) upgrades] pressures).
- Map stakeholders and existing work practices before the introduction of a new system—these are likely to span several organizational units in complex interventions.
- When evaluating an intervention, seek to assess both impacts and processes—this will allow assessment of how interventional elements were delivered.
- Remain flexible in delivering interventional components to fit in with busy work schedules—long training sessions are unlikely to be taken up.
- Encourage and document changes to interventional components over time—these are important to tailor the intervention to organizational and cultural contexts and therefore promote adoption.
- Compulsory interventional elements are likely to result in bigger impacts but may lead to resistance of users.
- Effective integration with existing technological systems can facilitate adoption—ideally, the system is integrated with existing EHRs.

Our work has implications for the implementation and evaluation of complex health care digitalization programs. Although stepwise implementations beginning with a limited number of sites may work in some instances where interventions have a limited impact, transformative complex interventions that integrate work processes of wider stakeholder groups may require a more integrated approach. Here, it is crucial that implementers scope existing work practices to understand in which settings the intervention needs to be launched. In our case, the Assessment Unit was an important location that had initially not been considered but was important for AMS, as many prescriptions were initiated in this setting.

Rather than simply seeking to measure impacts associated with an intervention, it is important to consider the processes that can deliver successful adoption and thereby use and impacts. For example, in relation to educational components, rather than envisaging a discrete period of formal prior face-to-face training, it may be worth considering training and awareness raising as ongoing activities that can be supported asynchronously and over an extended period that can be integrated into busy work schedules and chaotic working environments. Similarly, to increase awareness, there may be scope to provide visual prompts to increase awareness of intervention changes, for example with posters on every implementing ward including a shortened training video accessed via a QR code on the poster. Opportunistic approaches are likely to fit in better with competing priorities and pressures. However, there is a need to document all aspects of the intervention as it is delivered. This includes behavioral and educational activities and how these are delivered (and perhaps change) over time. This will help to determine the active intervention components and thereby help to understand delivery processes that impact effectiveness. Flexibility in delivery is often not desired by implementers, but we have illustrated that it is indeed necessary to help innovations embed within their socio-organizational environments. Although these changes are difficult to account for in quantitative studies, they need to be surfaced in qualitative process studies going forward to maximize the benefits of complex interventions.

Last, there is a need to recognize that developers of interventions are unlikely to ever be able to assess all user and contextual requirements upfront. As a result, interventions must be implemented and enhanced in real-life situations, which will need to involve process evaluation of early feasibility trials and iterative development of prototypes.

This evaluation had several limitations. It was implemented in a variety of wards but in only 1 hospital, which may not be representative of other institutions. In addition, it was performed soon after the implementation of the complex intervention, so the situation may not have been at steady state.

### Conclusions

In designing e-prescribing–based interventions that effectively support AMS, it is essential to acknowledge that, despite thorough planning, some requirements and obstacles to adoption may not be entirely foreseeable beforehand. It is also important to acknowledge that feasibility trials, although helpful at identifying potential issues, will not pick up all the requirements that will surface during full-scale implementation. A degree of trial and error is therefore necessary at all stages of implementation.

Developers and evaluators of complex interventions that include educational, behavioral, and technological elements in health care settings need to acknowledge that planned mechanisms are unlikely to be faithfully translated into practice.

Multiple technological, social, behavioral, and organizational factors influence the implementation and evaluation of digitalization initiatives, and these cannot always be fully anticipated. A degree of experimentation and agile refinement of intervention components is therefore necessary to mitigate potentially unanticipated negative consequences for safety and care delivery.

## Appendix

Multimedia Appendix 1 Quotes organized according to different types of interviewees.

## Abbreviations

AMR: antimicrobial resistance
AMS: antimicrobial stewardship
ePAMS+: electronic Prescribing-Based Antimicrobial Stewardship Plus
HIMSS: Healthcare Information and Management Systems Society
NHS: National Health Service
Opel: Operational Pressures Escalation
TPOM: Technology, People, Organizational, and Macroenvironmental

## Data Availability

Data are available on request.
