# Supplementary material for: Complex Hospital-Based Electronic Prescribing–Based Intervention to Support Antimicrobial Stewardship: Qualitative Study
Source: JMIR Form Res. 2024 Jul 26;8:e54458. doi: 10.2196/54458 (PMC11316148; doi:10.2196/54458)
Supplement: Multimedia Appendix 1 [file formative_v8i1e54458_app1.docx]

**Table S1. Quotes organized according to different types of interviewees**

| **Theme** | **Professional group** | **Quotations** |
| --- | --- | --- |
| **Difficulty planning for edge cases** | Consultants | I could foresee a few situations where it would not necessarily fit in with the intention of the antibiotic use, such as the antibiotics that are prescribed as a single one-off dose. So, we do that a lot in hematology and oncology, we use a single dose of gentamicin as part of our neutropenic sepsis treatment.  (Participant 1, Consultant, Hematology/Oncology, male)  The whole idea of a forty-eight-hour review is completely meaningless because it only ever is a one-off dose.  (Participant 1, Consultant Hematology/Oncology, male) |
|  | Pharmacist | What a focus group tells you and what a senior clinician tells you, and what a junior doctor wants is sometimes quite different, so we originally designed it with a link to the guidelines in but the senior clinician said there’s no point in having that because at that point you already know what antibiotic you want to prescribe so why would you need to go into the guideline but then…..I think what senior clinicians were thinking about that’s the initial decision…..but I think what they weren’t thinking about the purpose of the plan is to also allow review.  (Participant 7, Pharmacist/developer, male) |
|  | Microbiologist | N/A |
|  | Registrars (Specialty training 3-5) | The only difficulty is if you need to prescribe, say, two antibiotics at once, like amoxycillin and clarithromycin, you’ve got to do one, sign it off, and then do the other. For some reason, it won’t let you select two at once. (Participant 19, Registrar 3, Assessment Suite, female) |
|  | Speciality training 1-2 | Doxycycline, you often will give like loading dose, and then give a lower dose the next few days, but ePAMS will only prescribe the loading dose.  (Participant 15, Specialty Trainee Year 2, Respiratory, male) |
|  | Senior Nurse | N/A |
|  | Foundation Years 1-2 | like febrile neutropenia is kind of my bread and butter of what I would do and there’s a medication order set for that which prompts me to…if they’ve got a penicillin allergy these are the two I’m prescribing, which is just really nice and detailed and ePAMS doesn’t give me that. And I think because with febrile neutropenia we have to try tazocin or meropenem and gentamycin. With ePAMS, I think that gentamycin is getting missed and I think that’s dangerous for patient safety.  (Participant 10, Junior Doctor Foundation Year 2, Hematology/Oncology, female) |
| **Competing priorities: delivering an intervention whilst providing care** | Consultants | The first thing to say is that there’s been quite a lot of delays. I think, as always with these things, you start with the best intentions, but I think one of the major problems has been changes in eRecords over the… summer period.  (Participant 17, Consultant, Infectious Diseases, male) |
|  | Pharmacist | N/A |
|  | Microbiologist | N/A |
|  | Registrars (Speciality Training 3-5) | I’m aware of it. I believe it’s a tool to sort of try and promote antimicrobial stewardship, but I’ve not had any real sort of direct training or knowledge of it. (Participant 6, Specialty Trainee Year 5, Infectious Diseases, male) |
|  | Speciality trainees 1-2 | N/A |
|  | Senior Nurse | So [hospital] today and yesterday have had a bit of a crisis with the bedding situation. I’ve never known us be in this situation so bad where they’ve said, oh, we’re going to have to move people out.  (Participant 5, Senior Nurse, Infectious Diseases, female) |
|  | Foundation Years 1-2 | So I wouldn’t use it for that, especially if, you know, I’m stressed, I’m tired, if it’s out of hours, if someone’s really, really sick, I’m just going to do one, what my habit is, which is to go into medication list and two, just the safe option is to look for that order set so I know that I’m not going to miss anything. It’s going to prompt me and ePAMS doesn’t give me that. (Participant 8, Junior Doctor Foundation Year 1, Hematology/Oncology, female)  It's kind of a good thing and a bad thing. It's good for the stewardship, but bad if you're really busy and I think part of that might just be adapting and making the ePAMS review just part of your daily process. (Participant 3, Junior Doctor Foundation Year2, Infectious Diseases, male) |
| **Changing intervention components over time** | Consultants | The major one is that setting up the ePAMS in individual areas, probably works on [place] to some extent, but not in the other areas, because what has to happen is that the initial prescription needs to be done on ePAMS, and that would need to be done in the Assessment Suite, to then trigger the ePAMS happening on the base wards.  (Participant 17, Consultant, Infectious Diseases, male)  When the ePAMS plan comes, it gives you a kind of suggested dose or something like that, and probably the suggested dose for each of the antibiotics needs to be the most common one, or the one we would want people to prescribe most commonly, and that’s not quite the case at the moment, so that just needs tweaking.  (Participant 17, Consultant Infectious Diseases, male) |
|  | Pharmacist | We’ll get to a point where [name] will say, we’ve gone round all of the wards, they’re using the ePAMS plan, now just switch it on for everywhere, if that makes sense, once people have got a bit more familiar with it, then those fields will just become mandatory.  (Participant 7, Pharmacist/developer, male) |
|  | Microbiologist | N/A |
|  | Registrars (Speciality Trainees 3- 5) | It prompts the doctor during working hours, when it’s not the weekend, to review the antibiotics, that’s a huge advantage.. because there aren’t as many doctors around and therefore we wouldn’t want someone who’s not familiar with a patient to make that decision.  (Participant 13, Specialty Trainee 5, Assessment Suite, male) |
|  | Senior Nurse | N/A |
|  | Foundation Years 1-2 | I think if it was to go live, I appreciate that would be teething issues with any change, but the most effective way of everyone starting to… transferring over to it is to disable prescribing antibiotics through the other medication system. Taking it all off of that so you're forced to use that otherwise everyone, a lot of people will just, out of habit, go back.  (Participant 3, Junior Doctor Foundation Year 2, Infectious Diseases, male) |
| **Accounting for social and organizational transformations** | Consultants | Because the problem is if you don’t do that, the patient goes from here, the Assessment Unit, to Ward 52, and they haven’t had it done under ePAMS, that means Ward 52 have to re-prescribe under ePAMS, and by then it might be 24 to 48 hours into admission, so the antibiotic doesn’t get reviewed until three to five days instead of two to four days, or roughly, you know, two to three days, so that’s a bit of a problem. (Participant 1, Consultant, Hematology/Oncology, male) |
|  | Pharmacist | The only way that we would get that is if we said to the senior prescribers, you need to be the people that drive that change and tell them that they have to go in and do that and that becomes an expectation of the ward round.  (Participant 7, Pharmacist/developer, male) |
|  | Microbiologist | If they’re not doing the thing that we want them to be doing, then that’s probably an education issue, rather than a forcing them through pop-ups issue. (Participant 11, Microbiologist, male) |
|  | Registrars (Specialty training 3-5) | It prompts the doctor during working hours, when it’s not the weekend, to review the antibiotics, that's a huge advantage… because there aren't as many doctors around, and therefore, we wouldn't want, for instance, someone who's not familiar with a patient to have to make that decision. Also, it's a bit unfair too…because that will create a lot of work, increase the workload for a junior doctor. Because it'll probably be the [junior doctors] covering multiple wards to have to review ten, 20 antibiotics, and making a decision around antibiotics shouldn't really fall to the most junior person on the team.  (Participant 13, Specialty Trainee Year 5, Assessment Suite, male) |
|  | Specialty trainees 1-2 | I think obviously thinking of how it’s set up now, with it being very early on, the antibiotic choices are limited but my argument would be, they’re actually limited to all of the ones that we use often and if we were to give anything that isn’t on that list, we probably would be discussing it with infection diseases or micro anyway.  (Participant 20, Core Trainee 1, Assessment Suite, male) |
|  | Senior Nurse | N/A |
|  | Foundation Years 1-2 | I think one of the main problems is just clinical demand on time, and as in obviously prescribing it the usual way is a lot quicker, and sometimes it’s…you know, if you’re being pulled from pillar to post on the ward, on assessment suite, … it’s like ingrained in your behavior. It’s very easy to forget, and to just quickly prescribe 30 regimens, and then add the antibiotics just quickly on the same list through the same system, if that makes sense.  (Participant 16, Junior Doctor Foundation Year 1, Respiratory, male) |
